# Dimensional Accuracy Evaluation of Temporary Dental Restorations with Different 3D Printing Systems

**DOI:** 10.3390/ma14061487

**Published:** 2021-03-18

**Authors:** Wonjoon Moon, Seihwan Kim, Bum-Soon Lim, Young-Seok Park, Ryan Jin-Young Kim, Shin Hye Chung

**Affiliations:** 1 Department of Dental Biomaterials Science, Dental Research Institute, School of Dentistry, Seoul National University, 101 Daehak-ro, Jongno-gu, Seoul 03080, Korea; w.moon@snu.ac.kr (W.M.); nowick@snu.ac.kr (B.-S.L.); 2 Department of Aeronautical & Mechanical Engineering, Inha Technical College, 100 Inha-ro, Michuhol-gu, Incheon 22212, Korea; kseihwan@inhatc.ac.kr; 3 Department of Oral Anatomy, Dental Research Institute, Center for Future Dentistry, School of Dentistry, Seoul National University, 101 Daehak-ro, Jongno-gu, Seoul 03080, Korea; ayoayo7@snu.ac.kr; 4 Department of Dental Science, Dental Research Institute, School of Dentistry, Seoul National University, 101 Daehak-ro, Jongno-gu, Seoul 03080, Korea

**Keywords:** 3D printing, digital dentistry, temporary dental restoration, dimensional measurement accuracy

## Abstract

With the advent of 3D printing technologies in dentistry, the optimization of printing conditions has been of great interest, so this study analyzed the accuracy of 3D-printed temporary restorations of different sizes produced by digital light processing (DLP) and liquid crystal display (LCD) printers. Temporary restorations of 2-unit, 3-unit, 5-unit, 6-unit, and full-arch cases were designed and printed from a DLP printer using NextDent C&B or an LCD printer using Mazic D Temp (*n* = 10 each). The restorations were scanned, and each restoration standard tessellation language (STL) file was superimposed on the reference STL file, by the alignment functions, to evaluate the trueness through whole/point deviation. In the whole-deviation analysis, the root-mean-square (RMS) values were significantly higher in the 6-unit and full-arch cases for the DLP printer and in the 5-unit, 6-unit, and full-arch cases for the LCD printer. The significant difference between DLP and LCD printers was found in the 5-unit and full-arch cases, where the DLP printer exhibited lower RMS values. Color mapping demonstrated less shrinkage in the DLP printer. In the point deviation analysis, a significant difference in direction was exhibited in all the restorations from the DLP printer but only in some cases from the LCD printer. Within the limitations of this study, 3D printing was most accurate with less deviation and shrinkage when a DLP printer was used for short-unit restorations.

## 1. Introduction

Temporary restorations are temporary prostheses used to protect teeth under dental treatment or to replace lost teeth to stabilize the surrounding tissue and maintain esthetics. They protect the teeth from deformation, leakage, chemical irritation, and plaque accumulation before the final prosthesis is prepared [1]. In addition, the incorrect fabrication of temporary restorations may cause inflammation of the gingiva or alter the occlusion due to the movement of adjacent teeth [2], potentially causing serious issues with the placement of the final restoration. Therefore, it is essential to secure the safety and performance of temporary restorations.

Recently, with the rapid introduction of digital dentistry, 3D printing has been newly applied for the fabrication of dental prostheses [3]. Compared to the process of manufacturing temporary restorations through conventional means, 3D printing is considered more comfortable for patients and replaces much of the laboratory work [3]. In terms of efficiency, the digitalization of patient data allows for pre-planning, modification, and simulation during the procedure [4]. As the demand of 3D printing increases, various types of 3D printing techniques are being continuously developed, including digital light processing (DLP) and liquid crystal display (LCD).

The DLP technique is a method of producing a prosthesis through photo-polymerization by irradiating UV light to a tank containing acrylic or epoxy-based light-curing resin that reacts to ultraviolet rays (UV). The DLP technique is fast in printing and guarantees high precision. Therefore, it is used in the manufacturing of medical devices with complex or delicate shapes [5]. However, its biggest disadvantage is that it is expensive and limited to small-sized models. On the other hand, the LCD technique is also based on photo-polymerization but uses liquid crystals in imaging, rather than a light projector in DLP [6]. Compared to the other 3D printing techniques, the LCD technique is cheap and has good resolution, but has been known to have a short service life [7].

Although producing precise temporary restorative materials through 3D printing is critical, there have been a lack of studies regarding its accuracy. Few studies have been performed on 3D-printed temporary restorations, including the cytocompatibility of 3D-printed temporary restorations [8] and fabrication of highly viscous temporary crowns with fast building speed [9]. In addition, comparisons on the products of DLP and LCD-type printers have hardly been made. One study showed that DLP and LCD-type printers could print products of similar mechanical and biological properties if several adjustments in polymerization time and intensity were performed [6]. Therefore, it may be necessary to print temporary restorations using commonly used DLP and LCD techniques and study their accuracy. As the accuracy may vary according to size, variations in sizes may also be needed.

Composite resins have been widely used as restorative materials as they possess good physical, mechanical, and thermal properties [10]. To utilize them, extensive research has been performed on their reinforcement mechanisms [11,12,13,14]. In addition, there have been various successful methods to adjust for their esthetic properties such as translucency [15,16], staining [17], and shade [18]. In short, composite resins have various advantages and have been sufficiently studied for a long period of time. Thus, as long as the risk of cytotoxicity due to residual monomers is strictly controlled in the oral cavity [19], the use of composite resins for precise 3D printing may have numerous potential advantages.

Considering the importance of temporary restorations and the convenience of 3D printing technologies, the optimization of printing conditions may offer great advantages in the clinical environment. The accuracy analysis of 3D-printed restorations is one of the essential steps to expand the clinical use of 3D printers in the field of dentistry. Therefore, the purpose of this study was to analyze the accuracy of 3D-printed temporary restorations of different sizes produced by DLP and LCD 3D printers.

The null hypothesis was that the dimensional accuracy of 3D-printed temporary dental restorations would not be affected by their sizes and the type of printer.

## 2. Materials and Methods

### 2.1. 3D Model Fabrication and Scanning

**Figure 1:**
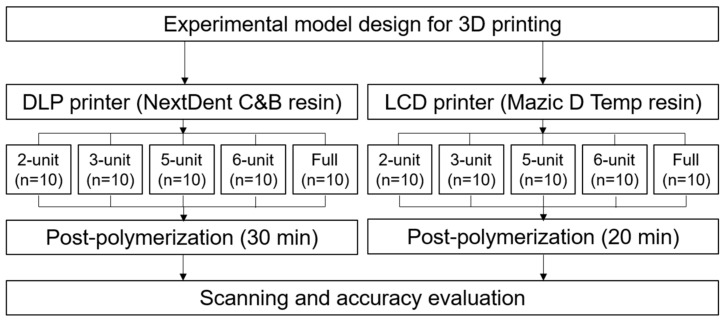
Flowchart of the experimental process.

**Figure 2:**
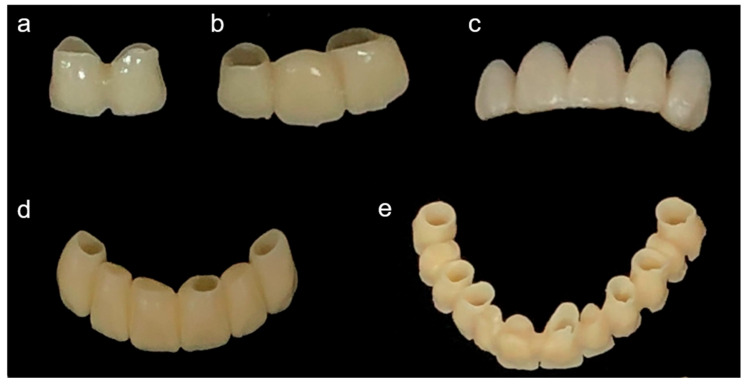
Representative printed restorations. (**a**) 2-unit, (**b**) 3-unit, (**c**) 5-unit, (**d**) 6-unit, and (**e**) full-arch restorations.

Temporary restorations of 2-unit, 3-unit, 5-unit, 6-unit, and full-arch cases were virtually designed from patient models in modeling software (Exocad DentalDB 2.2 Valletta, Exocad GmbH, Darmstadt, Germany) to produce reference STL files. Based on those models, each type of restoration was printed on a DLP-type printer (DLP; NextDent 5100, 3D Systems, Soesterberg, The Netherlands) and an LCD-type printer (LCD; Ka;rv LP550, Shinwon Dental, Seoul, Korea). The DLP printer utilized NextDent C&B (NextDent, Soesterberg, The Netherlands) and the LCD printer used Mazic D Temp (Vericom, Chuncheon, Korea) (Table 1). The DLP printer had a resolution of 1920 × 1080 pixels, build volume of 125 mm × 70 mm × 196 mm, 5 base layers, and post-polymerization time of 30 min (UV-A 315–400 nm; 72 W). The LCD printer had a resolution of 1440 × 2560 pixels, build volume of 68 mm × 121 mm × 140 mm, 5 base layers, and post-polymerization time of 20 min (UV-A 315–400 nm; 72 W). The printed restorations without any defects were selected by visual inspection (*n* = 10 each), washed with isopropanol, sonicated for five minutes, and dried at room temperature, according to the manufacturer’s instructions. The restorations were then post-polymerized (LC-3DPrint Box, 3D Systems, Soesterberg, The Netherlands) for 30 min (DLP) or 20 min (LCD), according to the manufacturer’s instructions. All the printed restorations were scanned using an optical scanner (MD-ID0300, Medit, Seoul, Korea) to obtain restoration STL files (Figure 1). The entire process was performed by an experienced single operator. Examples of the printed restorations are shown in Figure 2.

### 2.2. Superimposition and 3D Deviation Evaluation

**Figure 3:**
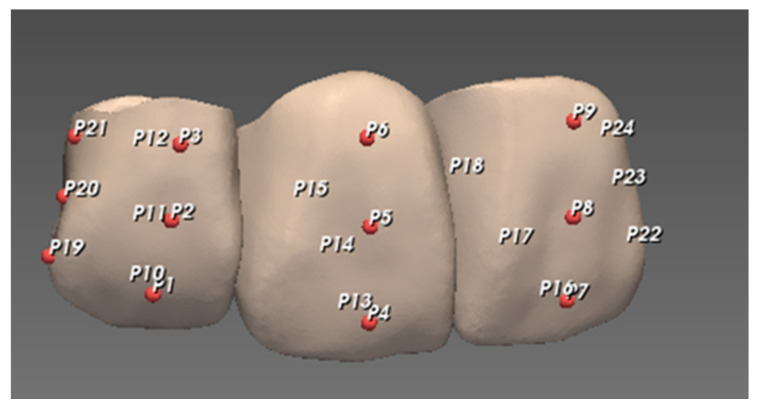
Representation of measuring points on 3-unit restoration: P1–P9 for buccal, P10–P18 for lingual, and P19–P24 for distal.

The entire three-dimensional external surface of the restorations was obtained using a 3D scanner that generated a point cloud (set of data points in space) for each restoration. Each restoration STL file was superimposed on the reference STL file to evaluate the trueness of the 3D-printed restorations using metrology software (PointShape Inspector v2.16, DREAMTNS, Seongnam, Korea). Superimposition was performed by “initial alignment” followed by “automatic alignment” functions. The deviation between the datasets of each restoration and the corresponding reference was calculated for all data points that consist of XYZ coordinates. The whole deviation was obtained to evaluate the displacement of the entire external surface and to generate an overall color map that indicates the direction of deviation with blue and red colors indicating negative and positive deviations compared to the reference, respectively. The sampling rate was set at 100%. With regard to the exclusion factor, the software evaluated the reference dataset and automatically set a “maximum distance value” above which it was excluded in the calculation. In addition to the whole-deviation analysis, specific measuring points were located on each reference restoration for point deviation analysis with 6 points on each unit—3 points on the buccal and 3 points on the lingual aspects of the unit, and an additional 3 points were assigned at the distal end of each restoration. Three points of each aspect were at the center of the coronal, middle, and cervical thirds, respectively (Figure 3). The median and interquartile range of the root-mean-square (RMS) and deviation values were calculated for trueness evaluation.

### 2.3. Statistical Analysis

For the whole-deviation analysis, a comparison among different restorations was performed by the nonparametric Kruskal–Wallis test followed by the Mann–Whitney test with Bonferroni’s correction. On the other hand, a comparison between different printers was performed by either the Student’s t-test or Mann–Whitney test depending on the normality test by the Kolmogorov–Smirnov test and Shapiro–Wilk test. For the point deviation analysis, a comparison among buccal, lingual, and proximal points was performed by the nonparametric Kruskal–Wallis test followed by the Mann–Whitney test with Bonferroni’s correction. The analysis was performed using statistical software (SPSS version 26.0, IBM, Armonk, NY, USA) under a significance level of 0.05.

## 3. Results

### 3.1. Whole-Deviation Analysis and Color Map

**Figure 4:**
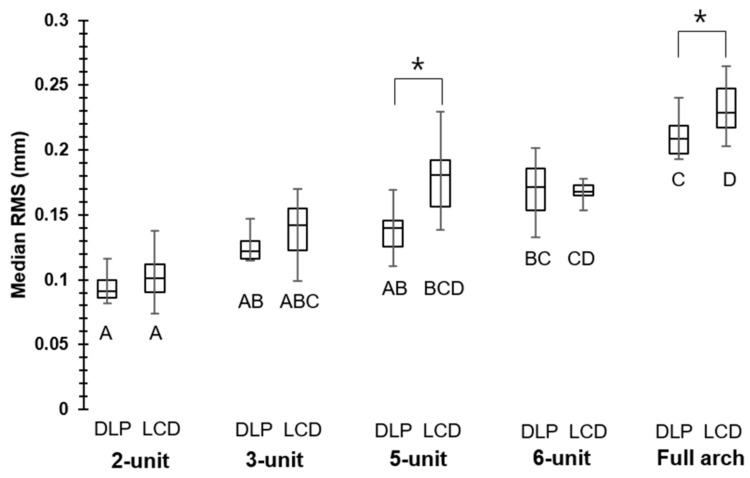
Median RMS of whole deviation; asterisks show significant difference within the same unit, whereas different uppercase letters (A–D) show significant difference within the same printers (*p* < 0.05).

**Figure 5:**
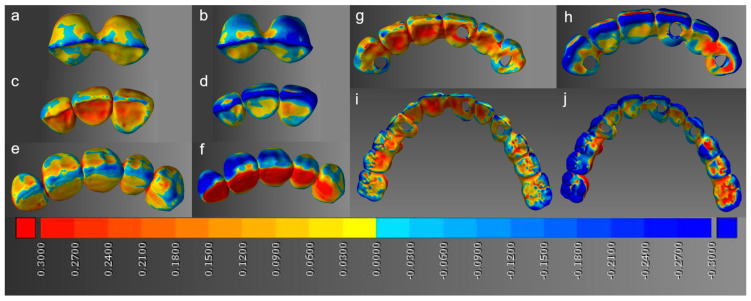
Color maps showing whole deviation; the blue and red colors indicate negative and positive deviations compared to the reference, respectively. (**a**) 2-unit DLP, (**b**) 2-unit LCD, (**c**) 3-unit DLP, (**d**) 3-unit LCD, (**e**) 5-unit DLP, (**f**) 5-unit LCD, (**g**) 6-unit DLP, (**h**) 6-unit LCD, (**i**) full-arch DLP, and (**j**) full-arch LCD restorations.

In the whole-deviation analysis, compared to 2-unit restorations, the RMS values were significantly higher in the 6-unit (0.17 mm) and full-arch (0.21 mm) cases for the DLP printer. On the other hand, they were significantly higher in the 5-unit (0.18 mm), 6-unit (0.17 mm), and full-arch (0.23 mm) cases for the LCD printer. A significant difference between the DLP and LCD printers was observed in the 5-unit and full-arch cases, where the DLP printer exhibited lower RMS values of 0.14 and 0.21 mm, respectively, compared to the LCD printer RMS values of 0.18 and 0.23 mm, respectively (Figure 4). Representative 3D images of the whole deviation are shown in Figure 5 as color maps. DLP restorations tended to exhibit less shrinkage (negative deviation) than LCD restorations, with the shrinkage being most remarkable on the occlusal surfaces.

### 3.2. Point Deviation Analysis

In the point deviation analysis, significant differences among the buccal, lingual, and proximal points were shown in all the restorations in DLP printing. Proximal deviations (0.06 mm) in the 2-unit cases, buccal and proximal deviations (0.11 and 0.07 mm) in the 3-unit cases, buccal and proximal deviations (0.06 and 0.14 mm) in the 5-unit cases, lingual deviations (0.13 mm) in the 6-unit cases, and proximal deviations (0.20 mm) in the full-arch cases were significantly higher within the same restorations. For LCD printing, significant differences were exhibited in the 5-unit, 6-unit, and full-arch cases, but not in the 2-unit and 3-unit cases. Lingual deviations (0.26 mm) in the 5-unit cases, proximal deviations (0.14 mm) in the 6-unit cases, and buccal and proximal deviations (0.13 and 0.18 mm) in the full-arch cases were significantly higher within the same restorations (Table 2).

## 4. Discussion

The null hypothesis that the dimensional accuracy of 3D-printed temporary dental restorations would not be affected by their sizes and type of printers was rejected. According to the whole-deviation analysis, the RMS increased with the restoration size. A significant increase in RMS value started to become noticeable in the 6-unit cases in DLP printing, whereas it began to appear in the 5-unit cases in LCD printing. Based on these results, it can be stated that DLP and LCD printing were both inaccurate in larger restorations. This might be attributed to the intrinsic limitation of DLP and LCD printers as they share the same basic operating mechanism. DLP and LCD printing can be used for small restorations, but 3D printers based on other technologies may be considered for the more accurate printing of larger restorations.

The performance of DLP and LCD printing differed in larger restorations such as the 5-unit and full-arch cases. In those cases, DLP printing exhibited lower RMS values, indicating that DLP printing showed better accuracy than LCD printing. However, in DLP and LCD printing, the important parameters are exposure time, wavelength, and amount of power supply [20]. As those parameters were not completely set to be the same for both printers, not enough evidence is available to evaluate the performance of DLP and LCD printing simply based on the results of this study. A comparison between DLP and LCD printing, as seen in this study, is very rare possibly due to their similar backgrounds. Previous studies have compared different 3D printing technologies such as stereolithography apparatus (SLA), DLP, fused filament fabrication (FFF), PolyJet technique, and fused deposition modeling (FDM) [21,22]. The analysis of DLP and LCD printing can be critical as the identification of advantages or disadvantages of one technique may allow for the complete replacement of one with another. This can be an important factor in terms of the efficiency of the research.

On the other hand, according to the point deviation, DLP printing exhibited differences in the direction of deviation in all the restorations. The deviation did not occur uniformly but in a skewed direction. However, LCD printing showed uniform deviation in all directions in the 2- and 3-unit cases but began to exhibit skewed deviations for larger restorations. As this result was not shown in the whole deviation analysis, it was found that the point deviation analysis was capable of revealing obscured data in the whole deviation analysis.

The present study utilized the 3D volumetric analysis in XYZ coordinates. In the past, however, linear measurement was widely used to determine the accuracy of dental models [23,24]. Linear measurement, while mostly based on limited measuring points, had an intrinsic limitation that it varied depending on the reference markers [25] and could not capture the 3D morphological changes [26]. 3D deviation measurement with color mapping as in this study was based on the point cloud in XYZ coordinates and was thereby expected to overcome the limitations of linear measurement.

As previously known, shrinkage is one of the challenges of DLP printers [27]. In the color map, such shrinkage was well-presented in both printers, and it was more prominent in LCD restorations. Although DLP and LCD printing are operated under similar mechanisms, their conditions for proper operation are different. For LCD printers to have similar mechanical properties to DLP printers, more powerful or longer post-polymerization processes are required [6]. In this study, there was no specific control on the post-polymerization scheme, so the LCD restorations may not have acquired sufficient mechanical properties to endure shrinkage. In addition, the shrinkage was more concentrated on the occlusal surfaces, and this was consistent with the previous studies that confirmed the occlusal surfaces to be more error-prone [28]. To use the printed restorations by DLP and LCD printing in the clinical environment, chairside adjustments of internal adaptions or designing larger STL files may be considered.

Shrinkage can also be analyzed in terms of the used materials. The shrinkage of composite resins is one of the most widely known characteristics. Numerous factors have been reported to be involved in the shrinkage of composite resins. For example, the differences in filler particles [29], degree of conversion [30], and curing methods [31] resulted in different shrinkage rates. Therefore, optimization of such conditions through changes in the printing parameters and printing materials may be needed to adjust for shrinkage. Moreover, as the printing materials used in this study were both based on methacrylic oligomers, the synthesis of low-shrinkage methacrylate monomers [32] to replace the original monomers may be another option.

Regarding dental models and surgical guides, there have been reports on their clinically acceptable accuracies [33,34,35]. In particular, less than 100 μm of difference from the reference was suggested as an acceptable value in dental models [36]. However, these results were based on a linear measurement of distances, so they may still have limitations. For the 3D-printed temporary restorations, such information is absent, so it is difficult to determine whether the inaccuracy that occurred in this study is within threshold values. Further studies are required to suggest clinically acceptable levels of inaccuracy.

This study had a limitation as an in vitro study. The accuracy of 3D-printed restorations should eventually be evaluated inside the oral cavity where additional considerations on temperature, moisture, and pH are required. For example, the current experimental conditions might be tested in vivo. Moreover, this study was confined to a limited number of printing techniques, printing materials, and build direction, and factors such as aging were also not considered. Although the printing materials in this study had the same overall chemical composition, they were from different manufacturers. As these factors are known to influence the accuracy of 3D-printed restorations [37,38], further studies are necessary to discuss the accuracy in more detail.

## 5. Conclusions

The 3D printing of temporary restorations was more inaccurate for larger restorations in both DLP and LCD printers. Differences between DLP and LCD printing were observed in larger restorations, where the degree of inaccuracy was smaller in DLP printing. The restorations by DLP printing were less prone to shrinkage, which was prominent on the occlusal surfaces. The direction of deviation was always skewed to one side in DLP printing, but was uniform in LCD printing for smaller restorations. Within the limitations of this study, 3D printing was most accurate with less deviation and shrinkage when a DLP printer was used for short-unit restorations. However, further studies to improve the degree of deviation, direction of deviation, shrinkage, and actual adaptation in the oral cavity are necessary.

## Figures and Tables

**Table 1:** Printing materials used in the study.

	NextDent C&B	Mazic D Temp
Printer	DLP	LCD
Shade	A3.5	A2
Chemical composition	Methacrylic oligomer, phosphine oxide	Methacrylic oligomer, phosphine oxide
Lot. Number	WY364N04	TP0961A2

**Table 2:** Median point deviation at the buccal, lingual, and palatal points.

		Median Point Deviation (mm)	
		DLP	LCD
2-unit	Buccal	0.04 [0.02, 0.07] ^A^	0.05 [0.03, 0.08] ^A^
	Lingual	0.04 [0.02, 0.06] ^A^	0.07 [0.03, 0.13] ^A^
	Proximal	0.06 [0.03, 0.12] ^B^	0.06 [0.03, 0.09] ^A^
3-unit	Buccal	0.11 [0.04, 0.17] ^B^	0.05 [0.02, 0.11] ^A^
	Lingual	0.04 [0.02, 0.09] ^A^	0.05 [0.02, 0.12] ^A^
	Proximal	0.07 [0.04, 0.13] ^B^	0.07 [0.02, 0.12] ^A^
5-unit	Buccal	0.06 [0.02, 0.12] ^B^	0.08 [0.03, 0.23] ^A^
	Lingual	0.04 [0.02, 0.07] ^A^	0.26 [0.09, 0.39] ^B^
	Proximal	0.14 [0.04, 0.21] ^C^	0.15 [0.11, 0.19] ^A^
6-unit	Buccal	0.08 [0.03, 0.16] ^A^	0.07 [0.02, 0.13] ^A^
	Lingual	0.13 [0.05, 0.23] ^B^	0.06 [0.03, 0.10] ^A^
	Proximal	0.08 [0.03, 0.17] ^A^	0.14 [0.05, 0.22] ^B^
Full arch	Buccal	0.09 [0.04, 0.17] ^A^	0.13 [0.05, 0.23] ^B^
	Lingual	0.10 [0.04, 0.19] ^A^	0.10 [0.04, 0.18] ^A^
	Proximal	0.20 [0.11, 0.28] ^B^	0.18 [0.12, 0.26] ^B^

Note: Absolute values were used for statistical analysis. Interquartile ranges [first quartile, third quartile] are shown in parentheses. Different uppercase letters indicate significant difference within the same restoration (*p* < 0.05).

## Data Availability

Data sharing is not applicable to this article.
